# Relationship between the lipid composition of maternal plasma and infant plasma through breast milk

**DOI:** 10.1007/s11306-019-1589-z

**Published:** 2019-09-25

**Authors:** Samuel Furse, Georgia Billing, Stuart G. Snowden, James Smith, Gail Goldberg, Albert Koulman

**Affiliations:** 1 Core Metabolomics and Lipidomics Laboratory, Wellcome Trust-MRC, Institute of Metabolic Science, University of Cambridge, c/o Level 4 Pathology, Addenbrooke’s Hospital, Cambridge, CB2 0QQ UK; 2 Nutrition and Bone Health Group, MRC Elsie Widdowson Laboratory, 120 Fulbourn Road, Cambridge, CB1 9NL UK; 3 Faculty of Mathematics & Physical Sciences, School of Food Science & Nutrition, University of Leeds, Leeds, LS2 9JT UK

**Keywords:** Candidate biomarkers, Lipids, Glycerides, Infant development, Breastfeeding

## Abstract

**Introduction:**

This study was motivated by the report that infant development correlates with particular lipids in infant plasma.

**Objective:**

The hypothesis was that the abundance of these candidate biomarkers is influenced by the dietary intake of the infant.

**Methods:**

A cohort of 30 exclusively-breastfeeding mother–infant pairs from a small region of West Africa was used for this observational study. Plasma and milk from the mother and plasma from her infant were collected within 24 h, 3 months post partum. The lipid, sterol and glyceride composition was surveyed using direct infusion MS in positive and negative ion modes. Analysis employed a combination of univariate and multivariate tests.

**Results:**

The lipid profiles of mother and infant plasma samples are similar but distinguishable, and both are distinct from milk. Phosphatidylcholines (PC), cholesteryl esters (CEs) and cholesterol were more abundant in mothers with respect to their infants, *e.g.* PC(34:1) was 5.66% in mothers but 3.61% in infants (*p *= 3.60 × 10^−10^), CE(18:2) was 8.05% in mothers but 5.18% in infants (*p *= 1.37 × 10^−11^) whilst TGs were lower in mothers with respect to their infants, *e.g.* TG(52:2) was 2.74% in mothers and 4.23% in infants (*p* = 1.63 × 10^−05^). A latent structure model showed that four lipids in infant plasma previously shown to be biomarkers clustered with cholesteryl esters in the maternal circulation.

**Conclusion:**

This study found evidence that the abundance of individual lipid isoforms associated with infant development are associated with the abundance of individual molecular species in the mother’s circulation.

**Electronic supplementary material:**

The online version of this article (10.1007/s11306-019-1589-z) contains supplementary material, which is available to authorized users.

## Introduction

Breastfeeding is generally regarded as the best start to a baby’s life. Babies who are breastfed typically have a healthy growth trajectory, suggesting that human milk provides the infant with the optimum diet for its development. Babies who are formula-fed can exhibit a weaker growth pattern and, when the process is managed poorly, can become malnourished (David and David 1984; Nisar et al. 2016). These and other considerations have led the WHO to advise that all children are breastfed exclusively for the first 6 months *post partum* (WHO 2002, 2009).

The differences observed in the growth trajectories of breast- and formula-fed babies may have a molecular origin. Recent evidence has shown that the circulation of breast-fed infants has a markedly different lipid and triglyceride profile compared to those fed formula milk (Koulman et al. 2014; Prentice et al. 2015). Evidence from animal models suggests that this may be due to nutritional programming. An animal model of diet-induced obesity and gestational diabetes mellitus (GDM) has shown that there is a profound effect on infants of their mother’s dietary intake and metabolism (Alfaradhi et al. 2016; Blackmore et al. 2014; Loche et al. 2018; Samuelsson et al. 2008).

**Fig. 1:**
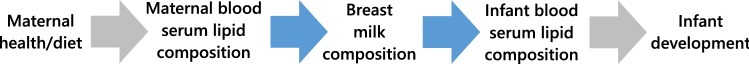
Schematic representation of the steps between the maternal diet and infant development

This is reflected in humans born small and large for gestational age due to exposures in utero, who are at increased risk of metabolic complications in later life (Tsadok et al. 2011; Wei et al. 2003). The infant circulation also reflects feeding and correlates with development in early childhood (Acharjee et al. 2017; Furse et al. 2019; Prentice et al. 2015), and hints that lipid metabolism is linked to infant development (Fig. 1) (Prentice et al. 2015). This raises questions about how dysregulation of metabolism later in life is associated with conditions in utero and in infancy, and how it might be detected early in life for appropriate intervention. This is consistent with long-standing evidence that the dietary intake of older children and adults affects the lipid and triglyceride composition of their blood plasma.

This mounting body of evidence led us to the general hypothesis that there is a relationship between the molecular composition (especially lipids, glycerides and sterols) of the maternal plasma, milk and her infant. Data published to date has provided evidence that the maternal diet is reflected in the composition of the milk produced (Brenna et al. 2007; Lassek and Gaulin 2014; Martin et al. 2012; Sabel et al. 2009) consistent with the considerable compositional variety in human milk (Gibson and Kneebone 1981; Stam et al. 2013). We therefore elected to test the relationship between the lipid composition of maternal plasma and infant plasma *through* the milk.

Specifically, we employed the hypothesis that the molecular profile of the mothers’ plasma may not only be dependent upon the direct influx (Fig. 1) but also shaped by metabolic processes. To test these hypotheses, we used the relative abundance of the five phospholipids in infant circulation that showed an association with weight and catch-up growth in our previous study, [PC(18:1/16:0) and PC-O(34:1)] and poor weight gain [PC(20:4/18:0), PC-O(36:4) and SM(d18:1/16:0)] (Prentice et al. 2015), in 30 Gambian infants.

Lipids were detected using high resolution mass spectrometry by direct infusion in both positive and negative ionisation modes in order to profile glycerides, sterols, zwitterionic lipids and anionic lipids. The relative abundance of each species identified was calculated separately for positive and negative ionisation modes. This approach built on work on the triglyceride profile of milks (Dugo et al. 2006) and early studies (Breckenridge and Kuksis 1967; Breckenridge and Kuksis 1968), but also allowed us to profile fats, lipids and sterols in order to explore their relationships as candidate biomarkers (CBMs) of growth. Each of the development lipids was cross-checked for a correlation with any lipid in the breast milk the infant received and then checked with the lipids in the mothers’ circulation.

## Methods

### Study design

This study was designed to determine which species if any in infant plasma are associated with the milk they are fed, and which species if any in the milk are associated with the mothers’ plasma. This necessitated an homogeneous group of participants with a similar diet, consistent sample collection, detailed measurement of molecular abundance and statistical analyses.

### Cohort

We used a set of 30 mother–infant pairs (meta-data in Table 1). This was a prospective observational cohort. Mothers were recruited during pregnancy from a single antenatal centre in The Gambia. The group was an homogenous one, individuals were not stratified according to disease, growth etc.

**Table 1:** Infant Z-scores were calculated using anthropometric measurements and WHO software, compared to international growth references (WHO)

Mothers (*n* = 30)	
Age (years)	25.6 ± 6.4
Parity median (range)	2 (1–9)
Height (cm)	162.8 ± 4.8
Weight (kg)	57.5 ± 6.7
BMI	21.7 ± 2.6
%fat	33.5 ± 5.9
Infants (*n *= 30)	
Age (weeks)	13.6 ± 1.8
% Male infants	47
Length (cm)	61.8 ± 4.9
Weight (kg)^a^	5.9 ± 0.9
Z-score ZWA^a^	− 0.4 ± 0.9
ZLA	0.2 ± 1.3
ZWL^a^	− 0.5 ± 1.5

*ZWA* weight-for-age Z-score, *ZLA* length-for-age Z-score, *ZWL* weight-for-length Z-score, *%fat* percentage maternal body fat, obtained by deuterium dose-to-mother

^a^No data from one subject

### Reagents and standards

Solvents were purchased from *Sigma*-*Aldrich Ltd* (Gillingham, Dorset, UK) of at least HPLC grade and were not purified further. Lipid standards were purchased from Avanti Polar lipids (Alabaster, AL; through Instruchemie, Delfzijl, NL) and used without purification. Consumables were purchased from Sarstedt AG & Co (Leicester, UK).

### Participants

Mothers attended morning clinics at *MRC Unit The Gambia at LSHTM Keneba* situated in the Kiang West region of Gambia (hereafter referred to as MRC Keneba) with their infants, April–August 2013. The meta-data of the participants is shown in Table 1. These subjects were chosen due to a relatively homogeneous maternal diet, breastfeeding practice, ethnicity, BMI and age range. All participants were healthy.

### Blood sample collection and processing

A sample of maternal plasma, milk and infant plasma were collected from each mother–infant pair (*n* = 30 pairs) within 24 h, and collected from all pairs between April and August 2013.

Blood was drawn by venepuncture from fasted individuals. Maternal fasted blood sampling times ranged from 09:03 to 11:45 h, and infant times ranged from 09:00 to 11:50 h. Infants were fed 0.20–2.65 h before venepuncture. Lithium Hepaparin plasma monovette tubes were placed on ice immediately and centrifuged (4 °C, 1800 × *g* for 20 min (HNR: Mistral 6000 Centrifuge, Sanyo Gallenkamp PLC., Leicester, UK; MRC Keneba: Centrifuge 5810R, Eppendorf, Stevenage, UK) within 1 h of collection. Centrifugation was repeated for slightly haemolysed samples. Lithium Heparin plasma aliquots were transferred to polypropylene microtubes (1.5 mL, Sarstedt AG & Co, Leicester, UK), and stored at − 80 to − 70 °C in the MRC Keneba laboratory until analysed (Keneba-SOP-4045; HNR-SOP-0367).

### Milk sample collection and processing

Milk was untreated fresh hindmilk (12.5–15 mL) that was collected in the morning between 07:28 and 12:20 h, within 24 h of venepuncture, and was stored at − 80 to − 70 °C. Samples were collected at the mother’s compound and transported to MRC Keneba. Fat was measured by the creamatocrit method (50–70 μL, 75 μL glass capillary, plugged with *Cristaseal*) on fresh samples (Lucas et al. 1978), after which aliquots (1 mL) were transported (− 78 °C) and stored − 40 °C. Samples were thawed on ice and centrifuged (13k × *g*, 15 min, HNR: Heraeus PICO 17 Centrifuge, Thermo Fisher Scientific Inc., Leicestershire, UK; MRC Keneba: Haematospin 1300, Hawksley & Sons Ltd., Sussex, UK). Breast milk fat concentrations were found to be normally distributed. There were no significant differences between days in breast milk fat (ANOVA adjusted for subject ID and study day number, Scheffe’s post hoc test, *p* = 0.5–0.9). The overall mean ± SD (range) of the concentration of fat in breast milk was 38.3 ± 15.0 (5.9–98.4) g/L.

### Extraction of the lipid fraction

The phospholipid, triglyceride and sterol fractions were isolated together using a method reported recently (Koulman et al. 2014). Briefly, plasma (25 µL) was injected, along with blank and Quality Control samples (QCs) in the wells of a glass coated 2.4 mL/well ninety-six-well plate (96w plate; Plate+™, Esslab, Hadleigh, UK). Water (100 μL, MilliQ) was added to each of the wells, followed by methanol (250 μL, HPLC grade, spiked with 0.6 μM 1,2-di-*O*-octadecyl–*sn*-glycero-3-phosphocholine, 1.2 μM 1,2-di-*O*-phytanyl–*sn*-glycero-3-phosphoethanolamine, 0.6 μM C_8_-ceramide, 0.6 μM *N*–heptadecanoyl-d-erythro-sphingosylphosporylcholine, 6.2 μM undecanoic acid, 0.6 μM trilaurin), followed by *tert*-butyl methyl ether (TMBE, 500 μL). The plates were then sealed (aluminium microplate sealing tape), agitated (10 min, 600 rpm) and centrifuged (10 min, 3.2k × *g*). A 96-channel pipette was used to transfer 200 μL of the organic solution to a glass-coated 240 μL 96w plate (Plate+™, Esslab, Hadleigh, UK). The plate was transferred to a Genevac EZ-2 evaporator (Genevac Ltd., Ipswich, UK) and dried. The samples were reconstituted (*tertiary*-butylmethyl ether, 25 μL and MS-mix [7.5 mM ammonium acetate in IPA:CH_3_OH (2:1)], 90 μL) and transferred to a glass-coated 384w plate and sealed immediately.

### Mass spectrometry

Samples were then direct-infused into an Exactive Orbitrap (Thermo, Hemel Hampstead, UK), using a Triversa Nanomate (Advion, Ithaca US). Samples were ionised at 1.2 kV. The Exactive started acquiring data 20 s after sample aspiration began. After 72 s of acquisition in positive mode the Nanomate and the Exactive switched to negative mode, decreasing the voltage to − 1.5 kV. The spray was maintained for another 66 s, after which the analysis was stopped and the tip discarded, before the analysis of the next sample began. Throughout the analysis the sample plate was kept at 10 °C. Samples were run in row order.

The phospholipid, sterol and glyceride signals obtained were used to calculate relative abundance of that variable (and were therefore ‘semi-quantitative’ signals) with the signal intensity of each variable expressed relative to the total lipid signal intensity after subtraction of blank samples and removal of signals that were greater than 8 ppm different from the expected (monoisotopic) mass. Separate calculations were made for signals acquired in the two modes. Some variable comprised two or more isobars (same *m/z*). Raw high-resolution mass-spectrometry data were processed using XCMS (www.bioconductor.org) and Peakpicker v 2.0 (an in-house R script). Only species that were measured in more than 5% of samples were included in further analyses. 1248 lipid signals were detected robustly using this method. The lipids have been identified as described previously (Acharjee et al. 2017; Furse and Koulman 2019; Koulman et al. 2014; Prentice et al. 2015) and the identification is at level 2 of the Metabolomics Standards Initiative.

### Statistical methods

Multivariate calculations were performed using Metabolanalyst 3.0 or 4.0 (Chong et al. 2018), univariate and bivariate comparisons were performed using Microsoft Excel 2013.

Univariate calculations were typically based on mean and standard deviation (±) or for the bivariate calculations a Student’s *T*-test was employed. Where relevant, significance thresholds were corrected for multiple variables *p*-value thresholds were based on the variables being dependent. Thus, thresholds were calculated based on 0.05 divided by the square root of the number of variables (1248 variables), giving a FDR threshold *p* value of 0.0014. In addition to Pearson correlation and Principal Component Analysis, a Latent Structure Model (LSM) clustering was employed that used the Bayesian Hierarchical Clustering (BHC) algorithm, a highly successful non-parametric multinomial Dirichlet Process (infinite mixture) model clustering tool (Heller and Ghahramani 2005; Savage et al. 2009) available as R/BHC in Bioconductor [available at https://www.bioconductor.org/packages/release/bioc/manuals/BHC/man/BHC.pdf].

## Results

**Fig. 2:**
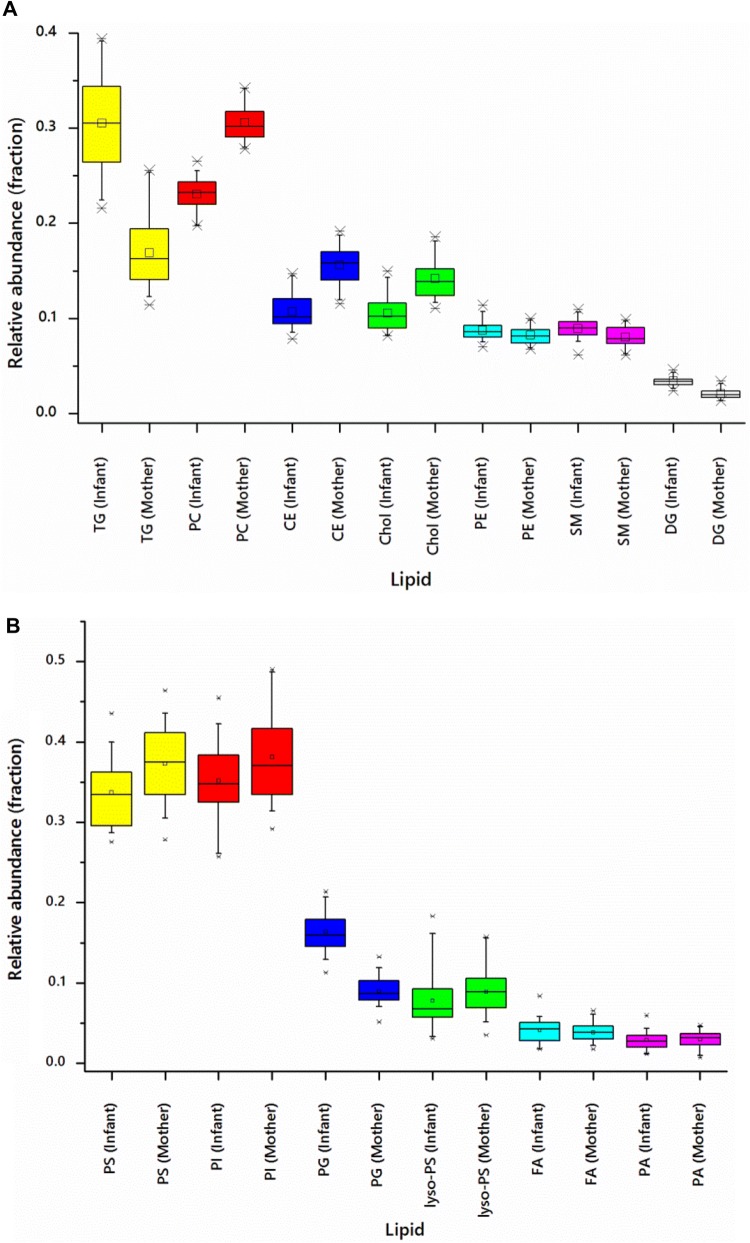
Box and whisker plots of the lipid profile of plasma samples from mother and baby pairs (n = 30), taken 3 months post partum. **a** Glycerides and zwitterionic lipids (+ve mode); **b** anionic lipids (−ve mode)

Mass spectra taken in positive ion mode indicated that triglycerides had a relative abundance twice as high in infant plasma as maternal (Fig. 2a, 30.527 ± 0.105% and 16.871 ± 0.068%, Table S1), with the abundance of cholesterol (Chol) and cholesteryl esters (CE) in infants being approximately 65% of that of their mothers. Phosphatidylcholines (PC) were around 20% more abundant in maternal plasma. Results from negative ion measurements indicated that phosphatidylinositols (PI) and phosphatidylserines (PS) were 10% less abundant in the infants’ plasma, however phosphatidylglycerols (PG) were approximately twice as abundant in infant plasma (Fig. 2b, 9.000% ± 0.330%, 16.371% ± 0.496%, Table S2).

**Fig. 3:**
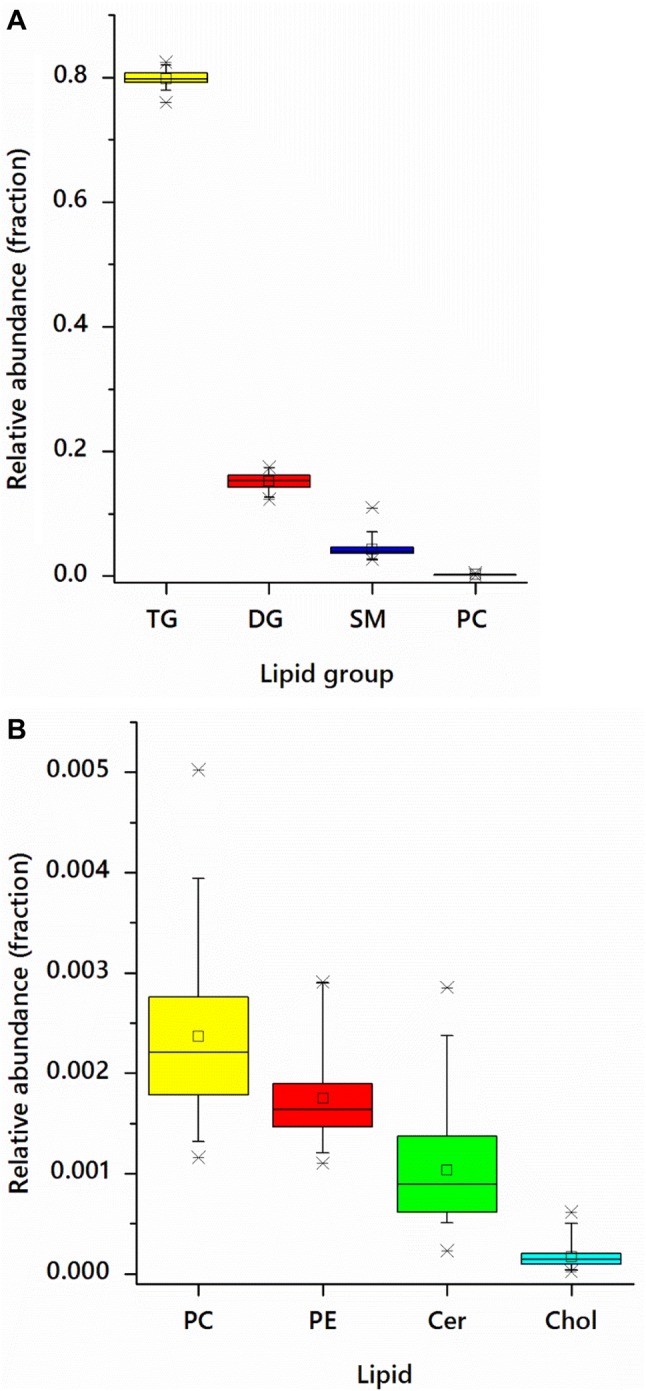
Box-and-whisker plots showing the lipid profile of breast milk samples from nursing mothers (n = 30), taken 3 months post partum. **a** Triglycerides (TGs), diglycerides (DGs), sphingomyelins (SMs) and phosphatidylcholines (PCs); **b** PCs, phosphatidylethanolamines (PEs), ceramides (Cer) and cholesterol (Chol). Spectra for both panels were taken in positive ion mode

Milk samples were dominated by glycerides, predominantly triglycerides (TGs, 80%) and diglycerides (DGs, 15–18%, fragments of TGs), Fig. 3a, Table S3, with sphingomyelins (SMs, ~ 4%) and phosphatidylcholines (PC ~ 0.25%) the most abundant phospholipids. Approximately 12.2% of the total (80% of the DG fraction) are *m/z* values associated with DGs that have lost one equivalent of water. Although this is well known to occur to ordinary DGs in the ionisation/gas phase used in mass spectrometry (Furse et al. 2015b), this fragment can also been ascribed to the TGs exposed to the same conditions. It is therefore not clear what proportion of the DG fraction can be ascribed to DGs of the sample, those produced by lipolytic activity on TGs during handling, or to the TGs of the milk as produced. However, as FAs only represent a minor component of the mass spectrum collected in negative mode (Table S4), we suggest that the bulk of the DG-H_2_O signals obtained originate from TGs.

Lower abundance species found in the positive mode include phosphatidylethanolamine (PE), ceramide and cholesterol (Fig. 3b). Profiling of the anionic lipids (taken in negative ion mode) suggested that PS was the most abundant (net) anionic lipid, with PI about a third as abundant (Table S4).

**Fig. 4:**
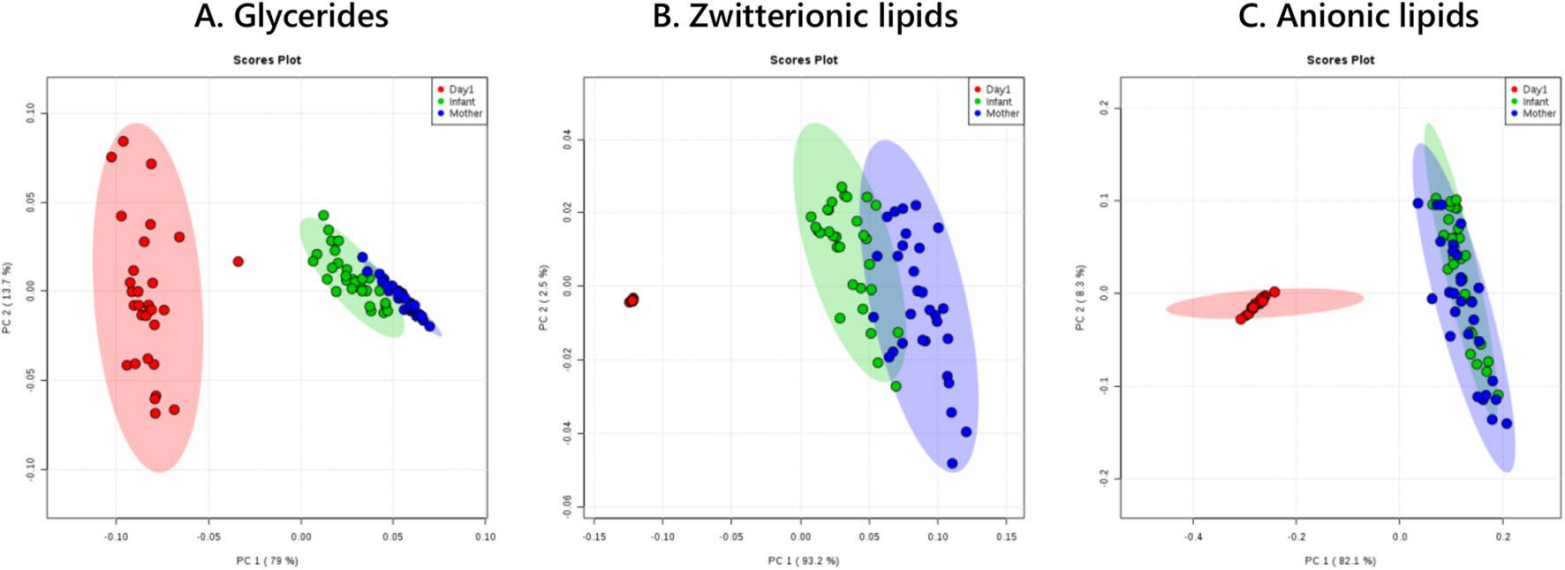
Principal component analyses of mothers’ and babies’ plasma samples and milk collected within a day of one another (n = 30) at 12 weeks post partum. **a** Mono-, di- and triglycerides (+ve ionisation mode); **b** zwitterionic lipids (+ve ionisation mode); anionic lipids (−ve ionisation mode)

Principal Component Analysis (Fig. 4) of the full lipid surveys showed that the mothers’ plasma was more similar to that of the infants’ and that both were distinct from the milk samples, despite this being the sole molecular connection between them and the infant’s only dietary source of fatty acids. There appears to be considerable variety in the glyceride fractions of milk samples (Fig. 4a) and in the zwitterionic lipids in plasma samples (Fig. 4b). However, the zwitterionic lipids in milk samples are remarkably contiguous (Fig. 4b) compared to differences observed in plasma. The profiles of anionic lipids follow the same general pattern (Fig. 4c).

Both mammary gland activity such as de novo lipogenesis and the digestion have a considerable effect on the profile of lipids and triglycerides in the process and probably only a small proportion of lipids will pass from the mother’s circulation to the infant’s circulation unaffected. This led us to examine how the CBMs for both healthy growth and poor growth in infant plasma may be connected to the flow of lipids and fats from the mothers’ circulation to the infants’ (Fig. 1). The composition of milk is notably different to either maternal or infant plasma, however it is undoubtedly the only dietary influencer of infant plasma lipids in exclusively breastfed infants.

We determined the correlations between recently-identified CBMs (Koulman et al. 2014; Prentice et al. 2015) for healthy growth [PC(18:1/16:0) and PC-O(34:1)] and poor weight gain [PC(20:4/18:0), PC-O(36:4) and SM(d18:1/16:0)] from the infants’ circulation with lipid and triglyceride species in the human milk they received. Variables with at least one Pearson correlation coefficient above 0.5 or below − 0.5 are shown in Table 2. These data indicate that the lipids associated with growth correlate with higher abundance of TGs with shorter carbon chains (42–46 carbons in the FARs) and with a lower abundance of TGs with longer carbon chains (54–57 carbons). The association between these and lipids in the mothers’ plasma were then calculated (Table 2). (A complete list of correlations between maternal plasma and human milk is shown in Fig. S1.)

**Table 2:**
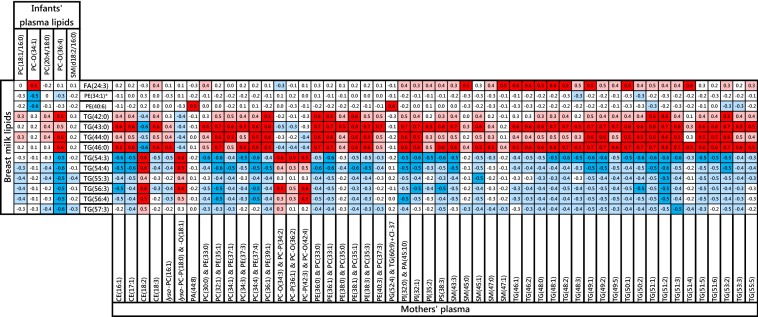
Pearson correlation coefficients of candidate biomarkers for infant development (top left), previously identified in infant plasma, with molecular species in the breast milk they are fed (left to right) and the correlations of the species identified in breast milk with molecular species in maternal plasma (lower right)

*Also comprises PC(31:1)

These data show that there is a relationship between the CBMs in infant plasma and several lipids and triglycerides in the milk consumed. As the lipids and fats in the maternal blood plasma supply the mammary glands for milk production, we developed the hypothesis that there would be a relationship between lipid profile of the breastfeeding mother’s blood plasma and the milk produced. Our data suggest a considerable number and broad range of lipids in the maternal plasma that may have a relationship with the milk lipid identified as being associated with infant growth (Table 2).

**Fig. 5:**
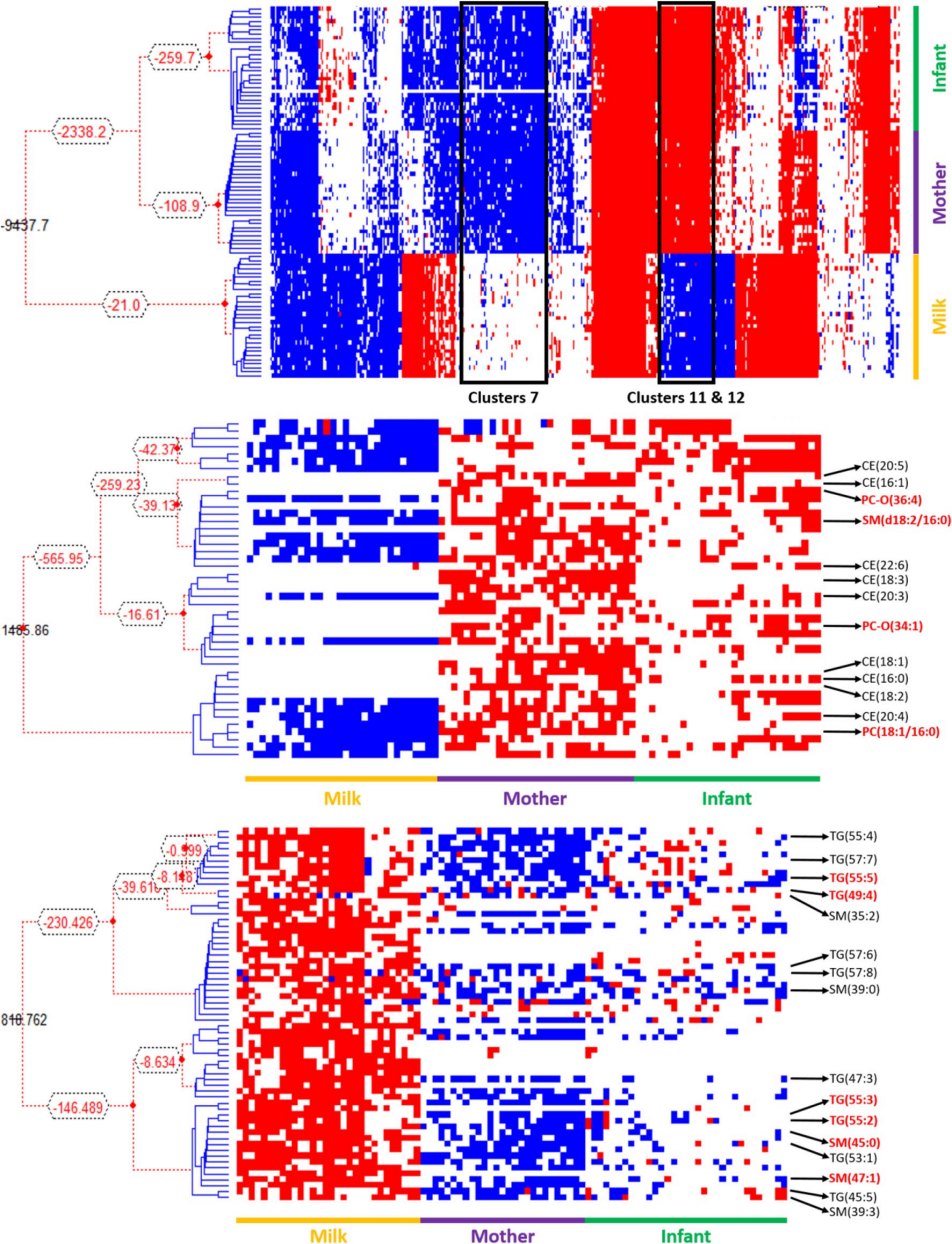
Latent Structure Model plots of the lipid profiles of maternal plasma, milk and infant plasma from a pilot cohort (n = 30) of mother-infant pairs from The Gambia. **a** Overall profile; **b** magnification of Cluster 7; **c** magnification of Clusters 11 and 12. Dendrograms indicate the hierarchy. Lipid isoform labels in red refer to growth biomarkers identified in previous work (**b**) or species comprising FARs with odd numbers of carbons that were also identified as candidate biomarkers through Pearson correlations (**c**). In **a**, the red is above the upperbound, white represents Marginal Likelihood, blue is below the lowerbound. Colours are inverted for **b** and **c** for clarity (Color figure online)

In order to identify the most important relationships between variables in the three samples and therefore the more important biomarkers, we used a Latent Structure Model (LSM) to explore the question of whether the lipid composition of maternal plasma is linked to that of the infant plasma through the molecular composition of the milk. This unsupervised approach is based on Bayesian hierarchical clustering (Heller and Ghahramani 2005; Savage et al. 2009) and in this application partitions analytes based on their abundance in the individuals and partitions individuals based on the abundance of the analytes. Figure 5a shows the relationships across the whole molecular survey for each sample. The LSM is an inference method for a Dirichlet Process Model (DPM) in which the prior probability that a given pair of clusters merge is defined by the DPM and determined solely by a concentration hyperparameter and the number of analytes or individuals respectively in each partition (Heller and Ghahramani 2005; Savage et al. 2009). The results (Fig. 5a–c) show that four of the five infant growth biomarkers appeared in one cluster (Cluster 11 and 12, Fig. 5b) which is surprising as a random chance calculation indicated that given that clusters 11 and 12 contain a total 46 lipid features only 0.54 of an infant growth marker should appear in this cluster by chance (i.e. ~ 1%). The fact that four of the five are seen together is an enrichment of 744%. We saw an enrichment of CE with 9 of the 11 species (an enrichment of 762%) co-clustering here. Cluster 7 saw an enrichment of odd-chain-containing triglycerides and SMs. Given that cluster 7 (Fig. 5c) contains 16 of the 65 odd-chain triglycerides and SMs and that there is a total 428 lipid features in total, then if the distribution of these species was being driven by chance we should only see 8.04 odd-chain species, demonstrating that we see an 216% enrichment (SM = 204% and triglycerides = 228%) in this cluster.

These data indicate that in this cohort of native Gambian women, all biomarkers appear but there is an association between four of the five, i.e. PC(34:1), PC-O(34:1), PC(38:4) and PC-O(36:4), SM(34:2). The data also imply that the species in milk with the strongest relationships with these candidate biomarkers and molecular species in the maternal circulation are TG(49:4), (55:2), (55:3), (55:5), SM(45:0) and SM(47:1) as they are identified by both methods. This is remarkable because it in indicates that odd-chain-containing FAs must be present in the milk of women whose diet contains very little dairy or other source of OCFARs.

## Discussion

The results in this paper detail the lipid profile of plasma samples from infants and their mothers, and fresh samples of the milk that passed between them within 24 h of collection of the plasma. The samples were taken from a cohort of 30 pairs between April and August 2013, a period that covered the end of the dry season and the beginning of the wet season, from a group of women with a roughly similar diet.

The abundance of lipid classes in milk (Tables S3 and S4) is reasonably consistent with previous work on the lipid and glyceride composition of this fluid (Bitman and Wood 1990; Bitman et al. 1983; Rodríguez-Alcalá and Fontecha 2010). However, the relatively high abundance of DGs, the reduced abundance of TGs, the normal abundance of SMs but low abundance of PCs suggests that the DG fraction is a composite of several sources, probably including lipases that are active in the milk (Bengtsson-Olivecrona and Olivecrona 1991; Deeth 2006; Neville et al. 1991) and during lipid collection (Furse et al. 2015a; Furse and Killian 2013), but mainly as an artefact of ionisation in mass spectrometry.

The proportions of phospholipids in the positive and negative ionisation modes are hard to compare due to poor cross-over and different ionisation efficiencies across the range and between modes. However a study of the phospholipid composition of bovine and ovine milk using phosphorus NMR (Murgia et al. 2003) showed that the ratio of PS to PI was ~ 1:9 and ~ 1:2.1, respectively. The proportions of PC, PE and SM were roughly the same in Cows’ milk, with PC around 80% of the abundance of SM and PE in Ewes’ milk. These data suggest that human milk is similar to these two, but with some distinct characteristics, and that negative ionisation mode is more similar to 31P NMR than to positive ionisation mode.

The Pearson coefficients calculated in the present analysis (Table 2) show that there are significant correlations between the CBMs for good and poor infant growth and certain TGs in the human milk, and between these TGs in the human milk and the profile of the mothers’ plasma. It is remarkable that a number of the TGs comprising 43–57 carbons, which is only possible through the presence of fatty acid residues with an odd number of carbon atoms (OCFARs), have emerged as CBMs. This suggests the incorporation of either or both C_15_ and C_17_ (margaric acid) FAs. Dairy foods can supply C_15_, however there is not a clear dietary source for C_17_, suggesting that it is produced endogenously. This is consistent with recent work indicates that the C_15_ found in humans has a dietary origin, where C_17_ is produced endogenously (Jenkins et al. 2017). As the supply of this results from the activity of the enzyme of the Hacl1 gene in humans, the question of whether there is a link between a reduction in the abundance of stearic acid and the supply of TGs in milk that are associated with infant development is raised. Further work using labelled analogues may offer insight into the precise role of these species in vivo.

Evidence for a role of the mother’s diet and health in the metabolic health of infants is accumulating (Aaltonen et al. 2010; Duque-Guimarães and Ozanne 2013; Fernandez-Twinn and Ozanne 2010; Linderborg et al. 2014) [review (Lönnerdal 1986)], and that of fathers is just beginning to emerge (Watkins et al. 2018). Specifically, the fatty acid residues (FARs) in the maternal milk are driven by FAs in the diet and the exchange of fats between the circulation and stores in the adipose and hepatic tissue, amongst other processes (Brenna et al. 2007; Lassek and Gaulin 2014; Martin et al. 2012; Sabel et al. 2009). On the other hand, myristic acid residues (14:0) are relatively abundant in the milk, which cannot come from the circulation. The evidence presented here supports the conclusion that there is extensive remodelling of the lipid and glyceride profile in the mammary glands, and again during infant digestion.

Recent work on the abundance individual lipid and glyceride species in human plasma has begun with direct infusion mass spectrometry in positive ionisation mode, detecting PCs, SMs, TGs, cholesterol and CEs (Koulman et al. 2014; Prentice et al. 2015). This work has shown that the abundance of several lipids changes as infants reach 12 months, and again after 12 months. Some isoforms of CE and TG only appear after 1 year, as do several isoforms of SM and PC (Koulman et al. 2014). Several commonplace isoforms also differ in abundance between formula- and breast-fed infants (Prentice et al. 2015). This suggests that the lipid profile of infants *in circulo* is affected by a number of exposures.

An unsupervised LSM (Fig. 5) was used as an orthogonal method to Pearson correlations in order to explore whether the same relationships between variables were observed in the global molecular profile. As this approach indicates that four of the five candidate biomarkers are clustered together (Cluster 7), and that several of the odd-chain-containing triglycerides cluster together (Clusters 11 and 12). We suggest that this is suitable evidence for further investigation of the role of these species in vivo.

Although we present the different sample types sequentially, based on the assertion that the plasma composition affects the milk composition and that in turn affects the circulating lipids of the infant, it cannot be excluded that all three lipid pools are also driven by other physiological processes that are responsible for these correlations. This is most likely for the correlations found between variables in the maternal plasma and milk as both samples are drawn from one individual. It is however relevant to determine the causes of the correlations observed as they offer a way of changing the lipid composition of the milk in a way that would affect the growth and development of the infant. This is important for situations where breastfeeding is not yielding a healthy growth trajectory. More generally, these data support the conclusion that there is a relationship between the lipid profiles at the various stages that shapes or even governs the supply of individual nutrients to infants, and thence the infant’s development.

## Electronic supplementary material

Below is the link to the electronic supplementary material.

Supplementary material 1 (XLSX 25 kb)

Supplementary material 2 (XLSX 9 kb)

Supplementary material 3 (XLSX 9 kb)

Supplementary material 4 (XLSX 9 kb)

Supplementary material 5 (XLSX 9 kb)

## Abbreviations

CBMs: Candidate biomarkers
CE: Cholesteryl ester
Chol: Cholesterol
DG: Diglyceride
FAR: Fatty acid residue
LSM: Latent structure model
OCFAR: Odd-chain-containing fatty acid residue
PA: Phosphatidic acid
PC: Phosphatidylcholine
PC-O: PC plasmalogen (sometimes known as alkyl-PCs)
PE: Phosphatidylethanolamine
PG: Phosphatidylglycerol
PI: Phosphatidylinositol
PS: Phosphatidylserine
SM: Sphingomyelin
TG: Triglyceride
WHO: World Health Organisation

## References

[CR1] Aaltonen J, Ojala T, Laitinen K, Poussa T, Ozanne S, Isolauri E (2010). Impact of maternal diet during pregnancy and breastfeeding on infant metabolic programming: A prospective randomized controlled study. European Journal of Clinical Nutrition.

[CR2] Acharjee A (2017). The translation of lipid profiles to nutritional biomarkers in the study of infant metabolism. Metabolomics.

[CR3] Alfaradhi MZ (2016). Maternal obesity in pregnancy developmentally programs adipose tissue inflammation in young, lean male mice offspring. Endocrinology.

[CR4] Bengtsson-Olivecrona G, Olivecrona T, Dennis EA (1991). Phospholipase activity of milk lipoprotein lipase. Methods in enzymology.

[CR5] Bitman J, Wood DL (1990). Changes in milk fat phospholipids during lactation. Journal of Dairy Science.

[CR6] Bitman J, Wood L, Hamosh M, Hamosh P, Mehta NR (1983). Comparison of the lipid composition of breast milk from mothers of term and preterm infants. American Journal of Clinical Nutrition.

[CR7] Blackmore HL, Niu Y, Fernandez-Twinn DS, Tarry-Adkins JL, Giussani DA, Ozanne SE (2014). Maternal diet-induced obesity programs cardiovascular dysfunction in adult male mouse offspring independent of current body weight. Endocrinology.

[CR8] Breckenridge WC, Kuksis A (1967). Molecular weight distributions of milk fat triglycerides from seven species. Journal of Lipid Research.

[CR9] Breckenridge WC, Kuksis A (1968). Specific distribution of short-chain fatty acids in molecular distillates of bovine milk fat. Journal of Lipid Research.

[CR10] Brenna JT, Varamini B, Jensen RG, Diersen-Schade DA, Boettcher JA, Arterburn LM (2007). Docosahexaenoic and arachidonic acid concentrations in human breast milk worldwide. The American Journal of Clinical Nutrition.

[CR11] Chong J (2018). MetaboAnalyst 4.0: towards more transparent and integrative metabolomics analysis. Nucleic Acids Research.

[CR12] David CB, David PH (1984). Bottle-feeding and malnutrition in a developing country: The ‘bottle-starved’ baby. Journal of Tropical Pediatrics.

[CR13] Deeth HC (2006). Lipoprotein lipase and lipolysis in milk. International Dairy Journal.

[CR14] Dugo P, Kumm T, Chiofalo B, Cotroneo A, Mondello L (2006). Separation of triacylglycerols in a complex lipidic matrix by using comprehensive two-dimensional liquid chromatography coupled with atmospheric pressure chemical ionization mass spectrometric detection. Journal of Separation Science.

[CR15] Duque-Guimarães DE, Ozanne SE (2013). Nutritional programming of insulin resistance: Causes and consequences. Trends in Endocrinology and Metabolism.

[CR16] Fernandez-Twinn DS, Ozanne SE (2010). Early life nutrition and metabolic programming. Annals of the New York Academy of Sciences.

[CR17] Furse S, Egmond MR, Killian JA (2015). Isolation of lipids from biological samples. Molecular Membrane Biology.

[CR18] Furse, S., Eriksen, K. G., Moore, S. E., Koulman, A. (2019). Identification of candidate molecular biomarkers for growth faltering in infants at 12w. In prep.

[CR19] Furse S, Killian JA (2013). Lipase activity in lipidomics—A hidden problem?. Molecular Membrane Biology.

[CR20] Furse S, Koulman A (2019). The lipid and glyceride profiles of infant formula differ by manufacturer, region and date sold. Nutrients.

[CR21] Furse S (2015). Synthesis of unsaturated phosphatidylinositol 4-phosphates and the effects of substrate unsaturation on SopB phosphatase activity. Organic & Biomolecular Chemistry.

[CR22] Gibson RA, Kneebone GM (1981). Fatty acid composition of human colostrum and mature breast milk. The American Journal of Clinical Nutrition.

[CR23] Heller, K. A., Ghahramani, Z. (2005). Bayesian hierarchical clustering. Paper presented at the Proceedings of the 22nd international conference on Machine learning, Bonn, Germany.

[CR24] Jenkins BJ (2017). Odd chain fatty acids: New insights of the relationship between the gut microbiota, dietary intake, biosynthesis and glucose intolerance. Scientific Reports.

[CR25] Koulman A (2014). The development and validation of a fast and robust dried blood spot based lipid profiling method to study infant metabolism. Metabolomics.

[CR26] Lassek WD, Gaulin SJC (2014). Linoleic and docosahexaenoic acids in human milk have opposite relationships with cognitive test performance in a sample of 28 countries. Prostaglandins, Leukotrienes and Essential Fatty Acids (PLEFA).

[CR27] Linderborg KM, Kalpio M, Mäkelä J, Niinikoski H, Kallio HP, Lagström H (2014). Tandem mass spectrometric analysis of human milk triacylglycerols from normal weight and overweight mothers on different diets. Food Chemistry.

[CR28] Loche E (2018). Maternal diet-induced obesity programmes cardiac dysfunction in male mice independently of post-weaning diet. Cardiovascular Research.

[CR29] Lönnerdal B (1986). Effects of maternal dietary intake on human milk composition. The Journal of Nutrition.

[CR30] Lucas A, Gibbs JA, Lyster RL, Baum JD (1978). Creamatocrit: Simple clinical technique for estimating fat concentration and energy value of human milk. British Medical Journal.

[CR31] Martin MA (2012). Fatty acid composition in the mature milk of Bolivian forager-horticulturalists: Controlled comparisons with a US sample. Maternal & Child Nutrition.

[CR32] Murgia S, Mele S, Monduzzi M (2003). Quantitative characterization of phospholipids in milk fat via P-31 NMR using a monophasic solvent mixture. Lipids.

[CR33] Neville MC, Waxman LJ, Jensen D, Eckel RH (1991). Lipoprotein lipase in human milk: Compartmentalization and effect of fasting, insulin, and glucose. Journal of Lipid Research.

[CR34] Nisar MU (2016). Feeding patterns and predictors of malnutrition in infants from poor socioeconomic areas in Pakistan: A cross-sectional survey. Cureus.

[CR35] Prentice P, Koulman A, Matthews L, Acerini CL, Ong KK, Dunger DB (2015). Lipidomic analyses, breast- and formula-feeding, and growth in infants. The Journal of Pediatrics.

[CR36] Rodríguez-Alcalá LM, Fontecha J (2010). Major lipid classes separation of buttermilk, and cows, goats and ewes milk by high performance liquid chromatography with an evaporative light scattering detector focused on the phospholipid fraction. Journal of Chromatography A.

[CR37] Sabel K-G, Lundqvist-Persson C, Bona E, Petzold M, Strandvik B (2009). Fatty acid patterns early after premature birth, simultaneously analysed in mothers’ food, breast milk and serum phospholipids of mothers and infants. Lipids in Health and Disease.

[CR38] Samuelsson A-M (2008). Diet-induced obesity in female mice leads to offspring hyperphagia, adiposity, hypertension, and insulin resistance. Hypertension.

[CR39] Savage RS (2009). R/BHC: Fast Bayesian hierarchical clustering for microarray data. BMC Bioinformatics.

[CR40] Stam J, Sauer PJ, Boehm G (2013). Can we define an infant’s need from the composition of human milk?. The American Journal of Clinical Nutrition.

[CR41] Tsadok MA (2011). Obesity and blood pressure in 17-year-old offspring of mothers with gestational diabetes: Insights from the Jerusalem Perinatal Study. Experimental Diabetes Research.

[CR42] Watkins AJ (2018). Paternal diet programs offspring health through sperm- and seminal plasma-specific pathways in mice. Proceedings of the National Academy of Sciences.

[CR43] Wei J-N (2003). Low birth weight and high birth weight infants are both at an increased risk to have type 2 diabetes among schoolchildren in Taiwan. Diabetes Care.

[CR44] WHO. (2002). *Infant and young child nutrition*. https://www.who.int/nutrition/topics/WHA55.25_iycn_en.pdf.

[CR45] WHO. (2009). *Infant and young child feeding: Model chapter for textbooks for medical students and allied health professionals*. https://apps.who.int/iris/bitstream/handle/10665/44117/9789241597494_eng.pdf. 23905206

